# Phage-Mediated Explosive Cell Lysis Induces the Formation of a Different Type of O-IMV in *Shewanella vesiculosa* M7^T^

**DOI:** 10.3389/fmicb.2021.713669

**Published:** 2021-10-08

**Authors:** Nicolás Baeza, Lidia Delgado, Jaume Comas, Elena Mercade

**Affiliations:** ^1^Secció de Microbiologia, Departament de Biologia, Sanitat i Medi Ambient, Universitat de Barcelona, Barcelona, Spain; ^2^Crio-Microscòpia Electrònica, Centres Científics i Tecnològics, Universitat de Barcelona (CCiTUB), Barcelona, Spain; ^3^Citometria, Centres Científics i Tecnològics, Universitat de Barcelona (CCiTUB), Barcelona, Spain

**Keywords:** membrane vesicles, gram-negative bacteria, explosive cell lysis, OMV, O-IMV, DNA, flow cytometry, electron microscopy

## Abstract

*Shewanella vesiculosa* M7^T^ is a cold-adapted Antarctic bacterium that has a great capacity to secrete membrane vesicles (MVs), making it a potentially excellent model for studying the vesiculation process. *S. vesiculosa* M7^T^ undergoes a blebbing mechanism to produce different types of MVs, including outer membrane vesicles and outer-inner membrane vesicles (O-IMVs). More recently, other mechanisms have been considered that could lead to the formation of O-IMVs derived from prophage-mediated explosive cell lysis in other bacteria, but it is not clear if they are of the same type. The bacterial growth phase could also have a great impact on the type of MVs, although there are few studies on the subject. In this study, we used high-resolution flow cytometry, transmission electron microscopy, and cryo-electron microscopy (Cryo-EM) analysis to determine the amount and types of MVs *S. vesiculosa* M7^T^ secreted during different growth phases. We show that MV secretion increases during the transition from the late exponential to the stationary phase. Moreover, prophage-mediated explosive cell lysis is activated in *S. vesiculosa* M7^T^, increasing the heterogeneity of both single- and double-layer MVs. The sequenced DNA fragments from the MVs covered the entire genome, confirming this explosive cell lysis mechanism. A different structure and biogenesis mechanisms for the explosive cell lysis-derived double-layered MVs was observed, and we propose to name them explosive O-IMVs, distinguishing them from the blebbing O-IMVs; their separation is a first step to elucidate their different functions. In our study, we used for the first time sorting by flow cytometry and Cryo-EM analyses to isolate bacterial MVs based on their nucleic acid content. Further improvements and implementation of bacterial MV separation techniques is essential to develop more in-depth knowledge of MVs.

## Introduction

Membrane vesicles (MVs) are produced by most bacteria and are involved in essential biological functions such as pathogenesis, inter- and intraspecies communication, biofilm formation, nutrient acquisition, and DNA transfer. In addition, they have great potential in immunology and biotechnology applications. For these reasons in recent years, numerous studies have investigated and reviewed all the aspects of bacterial MVs, including their composition, functions, biogenesis mechanisms, immunomodulatory capacity, and potential applications (Mashburn-Warren and Whiteley, 2006; Schooling and Beveridge, 2006; Lee et al., 2008; Kaparakis-Liaskos and Ferrero, 2015; Schwechheimer and Kuehn, 2015; Jan, 2017; Mozaheb and Mingeot-Leclercq, 2020).

Studies on membrane vesicles (MVs) in Gram-negative bacteria established an initial model that demonstrated how MVs formed from outer membrane protrusions solely contained the outer membrane and periplasmic material (Kadurugamuwa and Beveridge, 1996; Beveridge, 1999). These initially discovered MVs were named outer membrane vesicles (OMVs). However, increasing studies have highlighted how, depending on the mechanism of formation, Gram-negative bacteria give rise to different types of MVs having different structures and compositions. The different types of MVs and their origins have recently been discussed in detail (Gill et al., 2019; Toyofuku et al., 2019; Avila-Calderón et al., 2021).

*Shewanella vesiculosa* M7^T^, is a cold-adapted Gram-negative bacteria isolated from marine sediments of Antarctica (Bozal et al., 2009). Specifically, *S. vesiculosa* M7^T^ was named on the basis of its considerable capacity for producing MVs, which makes it a potentially excellent model for studying the vesiculation process (Frias et al., 2010). In previous studies, we demonstrated that *S. vesiculosa* M7^T^ produces a type of MV called outer-inner membrane vesicles (O-IMVs) containing two lipid bilayers (Pérez-Cruz et al., 2013). The O-IMVs are formed by the joint protrusion of the outer membrane and the plasma membrane with cytoplasmic content entrapped within the vesicles. These new types of vesicles are also found to be secreted by pathogenic Gram-negative bacteria (Pérez-Cruz et al., 2015). Although the roles played by stress factors such as antibiotics and environmental growth conditions in promoting vesiculation in many bacteria are already known (Mozaheb and Mingeot-Leclercq, 2020), no stress factors have been identified that help to produce blebbing O-IMVs in *S. vesiculosa* M7^T^.

More recently, other mechanisms have been considered that could lead to the formation of O-IMVs. Turnbull et al. (2016) demonstrated how explosive cell lysis through cryptic prophage endolysin activity can give rise to MV formation in *Pseudomonas aeruginosa* biofilms. They showed how the endolysin causes cell explosion leading to the fragmentation of the cell membranes that subsequent re-annealing, trapping different cytoplasmic components of the lysed cell. It is interesting to note that this explosive mechanism can give rise to both OMVs and O-IMVs. This model also holds in *Stenotrophomonas maltophilia* treated with ciprofloxacin, which leads to the induction of prophage and explosive cell lysis with the production of a pool of MVs including O-IMVs. These vesicles characteristically contain both outer and inner membranes and are enriched with cytosolic proteins (Devos et al., 2017). Recently, Mandal et al. (2021) demonstrated that bacteriophage infection of *Escherichia coli* generates different types of MVs through explosive cell lysis and membrane blebbing. The activation of prophages and their role in the maintenance of bacterial populations has been extensively studied; however, their importance as a mechanism of biogenesis of MVs is only now being considered (Catalao et al., 2012).

Prophage activation can be due to different stressors such as anoxia, UV radiation, iron depletion and antibiotic treatment among others (Kageyama et al., 1979; Bamford et al., 1987; Beaber et al., 2004; Binnenkade et al., 2014; Fang et al., 2017) and can trigger several morphological and physiological changes in bacteria that are commonly associated to apoptosis-like death (ALD) processes (Peeters and de Jonge, 2018; Mozaheb and Mingeot-Leclercq, 2020). Although prokaryotic ALD is currently a developing field, the morphological changes that have been observed are cell size and shape variation, chromatin condensation, DNA fragmentation and increased MV production (Andryukov et al., 2018). The physiological changes that have been observed are caspases activation, reactive oxygen species formation or metabolites levels alteration (NADH/NAD^+^, ADP/ATP or pyruvate, among others; Raju et al., 2006; Wadhawan et al., 2010). These stress responses can lead to the release of MVs enriched with stressed response proteins (Devos et al., 2017) or with a significantly higher amount of DNA (Andreoni et al., 2018), hence, prophage activation may play a key role in MV cargo and the functions that they may be involved.

The processes of blebbing or explosive lysis leading to O-IMV generation explain the different cytoplasmic components, such as nucleic acids, proteins, and ATP, found in MVs from various bacteria (Pérez-Cruz et al., 2013; Berleman et al., 2014; Kulkarni and Jagannadham, 2014; Turnbull et al., 2016; Cooke et al., 2019). Numerous studies have described the presence of DNA in MVs, but the mechanism by which it is packaged in the MVs remains largely unexplored (Dorward et al., 1989; Lotvall and Valadi, 2007; Rumbo et al., 2011; Biller et al., 2014, 2017; Bitto et al., 2017). The blebbing model for O-IMV could explain how plasmid and chromosomal DNA are packaged into MVs without cell death, specifically considering the existence of numerous mobile genetic elements capable of moving between plasmids or from a plasmid to the chromosome or vice versa (Partridge et al., 2018). On the contrary, the presence of heterogeneously sized DNA fragments in MVs from different regions of the chromosome points to O-IMV formation by mechanisms that cause cell death. These O-IMV formation mechanisms do not have to be mutually exclusive, although they do give rise to different types of O-IMVs.

Other relevant aspects to be considered are the growth stages in which O-IMVs are formed, and the proportion released to the media. It is unknown if the O-IMVs are produced during all stages of growth or if their formation is induced by stage-specific factors (Kuehn and Kesty, 2005; McBroom and Kuehn, 2007; MacDonald and Kuehn, 2013; Hagemann et al., 2014; Devos et al., 2017). Until now, O-IMV proportion estimations have mainly been based on Transmission Electron Microscopy (TEM) and Cryo-Electron Microscopy (Cryo-EM) observations. Using these methods, widely varying proportions of O-IMVs have been reported in different bacteria, such as 0.1% in *S. vesiculosa* M7^T^, 0.5% for *Pseudomonas* PAO1, 0.23% for *A. baumannii* AB41, or 49% in strains of *Pseudoalteromonas marina* (Pérez-Cruz et al., 2013, 2015; Hagemann et al., 2014; Devos et al., 2017).

In this study, we used flow cytometry with FM4-64 and SYBR^™^ Gold labeling to show that the various amounts and types of MVs produced by *S. vesiculosa* M7^T^ change during its growth. TEM after high-pressure freezing and freeze substitution (HPF-FS) of *S. vesiculosa* M7^T^ cultures revealed changes in the types of MVs during the different growth phases. The high resolution provided by TEM analysis confirmed the activation of a prophage-mediated explosive cell lysis leading to the formation of a different type of O-IMVs, and the production of MVs through re-annealing of ruptured membranes. We also sequenced MV DNA to characterize and map these nucleic acids. The heterogeneity of MVs produced point to the need to separate them for subsequent studies, for this reason, we specifically aimed to separate nucleic acid-containing MVs using flow cytometry-based sorting and Cryo-EM analysis.

## Materials and Methods

### Bacteria Used and the Growth Conditions

All studies were performed with *S. vesiculosa* M7^T^ (Bozal et al., 2009). For MV isolation and TEM observation, *S. vesiculosa* M7^T^ was grown in trypticase soy broth (TSB, Oxoid) using 2L baffled flasks filled with 500ml medium. Cultures were always inoculated at 1% with a 12h-incubated pre-inoculum. The flasks were shaken at 180rpm in an orbital shaker (Innova^®^ 44, New Brunswick Scientific) and incubated at 15°C. For MV analysis directly from supernatants and TEM, cytometry and apoptosis cell analysis, *S. vesiculosa* M7^T^ was grown in 500ml baffled flasks filled with 150ml of TSB (Oxoid) using the same culture conditions described above. When necessary, the growth was monitored by counting colony-forming units (CFU/ml) using the serial dilution method of plating on Trypticase soy agar plates.

### MV Isolation

*Shewanella vesiculosa* M7^T^ naturally secrete MVs into media. MVs were collected from the 500ml TSB cultures at different times-points (12, 18, 24, 48, 72 and 96h) using an adaption of the method described by McBroom et al. (2006). Cells were pelleted by centrifugation at 10,000×*g* for 30min at 4°C, and the supernatant was filtered through 0.45-μm pore-size filters to remove the remaining bacterial cells. MVs were obtained by centrifugation at 44,000×*g* for 1h at 4°C in an Avanti^®^ J-20 XP centrifuge (Beckman Coulter, Inc). The pelleted MVs were then resuspended in 50ml of Dulbecco’s phosphate-buffered saline (DPBS, Gibco, Life Technologies) and filtered through 0.22-μm pore-size Ultrafree spin filters (Millipore). Finally, the MVs were pelleted again at 44,000×*g* for 1h at 4°C and resuspended in a minimal volume of DPBS.

### High-Resolution Flow Cytometry for MV Analysis

Flow cytometry analysis of MV was performed as previously described by Wieser et al. (2014) with certain modifications. *S. vesiculosa* M7^T^ cultures were centrifuged at 10,000g for 30min, and 5ml of the supernatants were filtered through 0.22μm syringe filters (Puradisc^™^, Millipore) to analyze and quantify MVs in the supernatants. The supernatants were diluted in sterile DPBS to obtain the desired frequency of events per second (ev/s) between 1,000 and 10,000. The MVs were stained with the lipophilic fluorochrome FM4-64 (Invitrogen, T13320), resuspended in filtered-sterile DPBS at a final concentration of 0.5μg/ml, and incubated for 5min in the dark at room temperature (20–22°C). Sterile DPBS and sterile DPBS with FM4-64 were used as background controls. After incubation, controls and samples were analyzed with the BD FACS Aria^™^ Fusion II cytometer (BD Biosciences). The analyses were carried out for 30s at a flow rate of 2 with a trigger on FM4-64 fluorescence.

FM4-64 and SYBR^™^ Gold (Invitrogen, S11494) labeling were carried out to detect MVs with internalized nucleic acids. The SYBR^™^ Gold labeling was done using a 1/10,000 dilution of the stock solution, and the samples were incubated for 15min at room temperature in the dark. Samples treated with sterile DPBS, sterile DPBS with FM4-64, and sterile DPBS with FM4-64 and SYBR^™^ Gold were used as controls, and a fluorescence threshold was established.

A relation between the number of cytometer events and the number of MVs was established using FluoSpheres^™^ Carboxylate-Modified Microspheres (Life Technologies, F8803), with a size of 100nm and a concentration of 3.63×10^13^ microspheres/ml, to quantify MVs by flow cytometry. The FluoSpheres^™^ were diluted in filtered-sterile DPBS to obtain frequencies of events/s between 1,000 and 10,000 to quantify the isolated MVs. The FluoSpheres^™^ were diluted in filtered-sterile TSB, in the same way to quantify MVs from bacterial culture supernatants.

### Sorting of MVs by High-Resolution Cytometry

MVs were isolated and stained with both SYBR^™^ Gold and FM4-64 to separate MVs from those only stained with FM4-64 to identify the ones with internalized nucleic acids. A 70-μm nozzle was used on the BD FACS Aria^™^ Fusion II cytometer (BD Bioscience) for separation. First, the controls (sterile and filtered DPBS, sterile and filtered DPBS with FM4-64, and sterile and filtered DPBS with FM4-64 and SYBR^™^ Gold) and samples to be separated were analyzed to verify the percentage of events detected to have both fluorochrome labels and to identify the range of events/s. Then, two sterile 15ml collecting tubes (TPP^®^, Merk) containing 100μl of sterile water were used to collect each separated sample. PBS FacsFlow^™^ was used as the dilution buffer used during the sorting process (Fischer Scientific, United Kingdom). Once the sorting started, the injection flow was monitored and adjusted to keep the separation efficiency above 85%. The collected MVs were transferred to 8ml polycarbonate centrifuge tubes (Beckman Coulter) and centrifuged in the OPTIMA^™^ L-90K ultracentrifuge (Beckman Coulter) with Ti/70 rotor at 100.000xg for 90min at 4°C. Supernatants were discarded, and pellets were resuspended in 20μl of sterile water and kept at 4°C until fixation for Cryo-EM.

### TEM Observation After HPF-FS

TEM observation of *S. vesiculosa* M7^T^ was performed as described previously (Pérez-Cruz et al., 2015) with some modifications. Briefly, liquid cultures at different incubations times were centrifuged at 40,000×*g* for 1h at 4°C and cryo-immobilized using a Leica HPM100 high-pressure freezer (Leica Microsystems, Vienna, Austria) to observe the cells and MVs simultaneously. The cryo-immobilized samples were then freeze-substituted in pure acetone containing 2% (w/v) osmium tetraoxide (EMS, Hatfield, United States) and 0.1% (w/v) uranyl acetate (EMS, Hatfield, United States) at −90°C for 72h in an EM AFS2 (Leica Microsystems, Vienna, Austria). Then, they were warmed up to 4°C at a 5°C/h slope, kept at 4°C for 2h, and then kept at room temperature for 2h in darkness. The samples were washed in acetone at room temperature, infiltrated in increasing concentrations of Epon-812 resin (Epon 812, Ted Pella, Inc., United States) in acetone till pure Epon-812 was obtained. Finally, the samples were embedded and polymerized in Epon-812 at 60°C for 48h. Ultrathin sections (50–60nm) were obtained with a UC6 ultramicrotome (Leica Microsystems, Vienna, Austria) and placed on Formvar coated copper grids. The sections were stained with 2% (w/v) uranyl acetate for 30min and lead citrate for 5min. The samples were examined in a Tecnai Spirit microscope (FEI, Eindhoven, Netherlands) equipped with a tungsten cathode. Images were captured at 120kV with a 1,376×1,024-pixel CCD camera (FEI, Eindhoven, Netherlands).

### Cryo-EM Observation of Isolated MVs

The MVs were cryoimobilized using the Plunge Freezing technique (Delgado et al., 2019) for Cryo-EM visualization. The cryo-immobilization was performed in the Vitrobot Mark III (FEI, Eindhoven, Netherlands). One 3μl drop of the suspension was applied on the carbon surface of a glow-discharged Lacey Carbon 300 mesh copper grid (Ted Pella, United States) and held for 1–4min at 100% humidity. The excess liquid was automatically blotted with filter paper, and the sample was immediately plunge-frozen in liquefied ethane. The vitrified sample was then transferred to a Tecnai F20 EM (FEI, Eindhoven, Netherlands) using a cryo-holder (Gatan, Pleasanton, United States). The visualization of samples was carried out at 200kV, at temperatures between −180 and−170°C and at low-dose image conditions. The images were acquired with a 4,096×4,096-pixel CCD Eagle camera (FEI, Eindhoven, Netherlands). The quantification and subsequent analyses of the different MVs were carried out with the ImageJ program (Schindelin et al., 2012).

### Analysis of Phosphatidylserine Exposure

The Annexin V-FITC Apoptosis Detection Kit (Sigma) was used to identify the amount of exposed phosphatidylserine of *S. vesiculosa* M7^T^ cells at different times of growth to investigate apoptosis. Two 1ml aliquots of each sample were centrifuged at 10,000g for 10min at 20°C and washed twice in DPBS. Cells were resuspended and diluted to 10^6^ cells/ml in 1×Binding Buffer (BB). The Annexin V-FITC was added to each sample at a final concentration of 0.5μg/ml. Propidium iodide (PI) was also added to the samples at a final concentration of 2μg/ml, and they were incubated for 10min at room temperature in the dark. Stained cells were analyzed with the BD FACS Aria^™^ Fusion II cytometer (BD Bioscience). Several negative controls were used to optimize the analyses: 1×BB, 1×BB with both the fluorochromes, and 1×BB with *S. vesiculosa* M7^T^ cells without fluorochromes. This experiment was carried out with each condition in duplicates in four independent experiments.

### DNA Fragmentation Assay

TheAPO-BrdU^™^ TUNEL Assay (Invitrogen) was performed on the bacteria to analyze their cellular DNA damage at different times of growth. Two 1ml aliquots of each sample were diluted to 10^7^ cells/ml in DPBS in a final volume of 500μl. The bacterial suspension was added to 4.5ml of 2% (w/v) paraformaldehyde and incubated for 15min on ice. The cells were then centrifuged at 10,000×*g*, at 20°C and the supernatant was discarded. The pellet was resuspended in DPBS and washed twice. The cells resuspended in 100μl of DPBS were added to 900μl of 70% (v/v) ethanol and kept at −20°C for 12h. The alcohol suspension was centrifuged at 10,000*g* for 5min to remove the alcohol, and the pellet was resuspended in 50μl of the labeling solution. The cells were incubated at 37°C in the dark for 4h and shaken every 15min. Washing buffer (450μl) was then added and centrifuged at 10,000g for 5min. The pellet was resuspended in 100μl of the kit antibody solution. The cells were incubated at room temperature in the dark for 30min. A total of 500μl of the staining solution containing RNase A and PI was added to the resuspended cells and incubated at room temperature in the dark for 30min. Cell analysis was performed on the BD FACS Aria^™^ Fusion II cytometer (BD Bioscience). This experiment was carried out with each condition in duplicates in two independent experiments.

### Determination of Intracellular NAD(H) Levels

A colorimetric assay kit for NAD+/NADH Quantification (Sigma-Aldrich) was used to determine the intracellular levels of NADH and NAD+. Two 1ml aliquots were collected from each bacterial culture and were centrifuged at 10,000*g* for 10min and washed twice in DPBS. Extraction of the cytoplasmic material from the *S. vesiculosa* M7^T^ cells was carried out using the perchloric acid method (Bagnara and Finch, 1972) without further purification of the NAD+ and NADH molecules. Detection of total NAD (H) and only NADH was carried out according to the manufacturer’s instructions. This experiment was carried out with each condition in duplicates in three independent experiments.

### Determination of Intracellular ATP Levels

Two 1ml aliquots of *S. vesiculosa* M7^T^ liquid cultures were centrifuged at 10,000*g* for 10min and washed twice in DPBS. The cytoplasmic material of the cells was extracted using the perchloric acid method (Bagnara and Finch, 1972). Then the intracellular ATP levels were measured using the BacTiter-Glo^™^ Microbial Cell Viability Assay Reagent kit (Promega). The experiment was carried out with each condition in duplicates in three independent experiments.

### Extraction and Sequencing of DNA Coming From the MVs

Isolated MVs were pre-treated with DNase I (2U/μl, 1h, 40°C; Thermo Scientific), diluted 1/100 in sterile DPBS, and re-pelleted (44,000×*g* for 1h at 4°C) to remove the DNAse I. The amount of MVs was quantified by the Purpald method (Lee and Tsai, 1999). The DNA from the MVs was extracted with the PureLink^™^ Microbiome DNA Purification kit (Invitrogen). The extraction was performed from 50μl of MVs resuspended in DPBS with an LPS concentration of 0.5μg/μl. The extracted DNA was eluted with 50μl of the elution buffer. Quantification of the DNA extracted from the MVs from *S. vesiculosa* M7^T^ was carried out with the Quant-iT^™^ PicoGreen^®^ dsDNA kit (ThermoFisher Scientific). A phage *λ* DNA standard curve was established with 1:10 serial dilutions from 0.05ng/ml to 50ng/ml. TE buffer (10mm TRIS-HCl, 1mm EDTA, pH 7.5) was used as the negative control, and a genomic DNA sample of *S. vesiculosa* M7^T^ of known concentration was used as a positive control. Fluorescence measurement was carried out in the Modulus microplate reader (Turner Biosystems) with an excitation of 485/20nm and an emission of 528/20nm. Three independent DNA extraction experiments were performed, and DNA was measured in duplicate for each sample.

Libraries preparation from the DNA samples extracted from the MVs of *S. vesiculosa* M7^T^ was done using the NEBNext^®^ Ultra DNA Library Prep kit (Illumina). For each sample, the DNA was end-repaired, adenines were added to the 3' end, and the NEB adapters were ligated. The DNA bound to the adapter was cleaned in two steps to select both small and large fragments. A first incubation was carried out with 0.75×AMPure Beads (Beckman Coulter) to obtain the fraction with the large fragments. Then, the supernatant was purified with 1.1×AMPure Beads (Beckman Coulter) to obtain the fraction with the small fragments. Both fractions were separated into two aliquots: one (10%) to determine the number of amplification cycles up to the plateau phase using real-time PCR, and the other (remaining 90%) to carry out the PCR with the amplification cycles already optimized. This PCR was done using the NEBNext^®^ Multiplex Oligos for Illumina in both aliquots. Final libraries were analyzed using Agilent Bioanalyzers (Agilent, Germany) to estimate the quantity and size distribution of the quantified PCR products using the KAPA Library Quantification kit (KapaBiosystems) before amplification with Illumina’s cBot. This method allowed library preparation from low amounts of DNA and separation of the two fractions according to the size of the DNA fragments. Once the library was prepared, it was sequenced using the HiSeq 2500 System (Illumina), generating paired-end reads of 125bp in length. The entire sequencing process was monitored using the Sequencer Software HiSeq Control Software 2.2.58.

### *S. vesiculosa* M7^T^ Genome Sequencing

DNA from *S. vesiculosa* M7^T^ cells was prepared for subsequent sequencing using the Illumina DNA prep kit (Illumina). Once the library was prepared, it was sequenced using the HiSeq 2500 System (Illumina), generating 125bp long paired end reads with each fragment generated in the library being sequenced from both ends. The entire sequencing process was monitored using the Sequencer Software HiSeq Control Software 2.2.58. The genome of the strain was also sequenced using the Nanopore system. The library preparation kit used for the DNA extracted from *S. vesiculosa* M7^T^ cells was SQK-LSK109 (Nanopore Systems) to obtain the assembled and recircularized *S. vesiculosa* M7^T^ genome by using the Flongle FLO-FLG001 flow cell (Nanopore Systems). The monitoring and capturing of the sequencing results were performed using the main MinKnow software (v3.6.0) and the Bream software (v4.3.12). First, base-calling was performed to obtain the reads resulting from sequencing using the Guppy software (v3.2.8). Minimap2 was used (Li, 2016) to align the nucleotide sequences and correct possible errors. For the assembly of the *S. vesiculosa* M7^T^ genome, Miniasm software was used (Li, 2016). Finally, Racon (v1.4.13, Vaser et al., 2017) was used to correct raw contigs generated polish the final genome after assembly. Additionally, to further correct assembly errors, the reads from the previous Illumina sequencing studies were mapped to the new assembly using bowtie2 (v 2.3.0, Langmead et al., 2019) and analyzed using Pilon (Walker et al., 2014) to correct the assembly errors. Once the genome was assembled, the genes were predicted using the Quast tool (v5.0.2, Gurevich et al., 2013) and subsequently identified using the Glimmer tool (v3.02, Delcher et al., 2007) that distinguishes coding from non-coding regions in bacterial genomes. The genes identified to code for proteins were translated into amino acid sequence by transeq (v24) and aligned with the Uniref90 database by Diamond (v0.9.24, Buchfink et al., 2015). The closest match to the database was kept as the most likely protein description for each estimated gene. PHASTER tool (PHAge Search Tool Enhancer Release, Arndt et al., 2016) was used to identify potential phage presence in *S. vesiculosa* M7^T^ genome. The assembled genome was input in the FASTA format.

### *In silico* Analysis of the DNA From MVs of *S. vesiculosa* M7^T^

After sequencing the DNA fragments from *S. vesiculosa* M7^T^ MVs, sequences of the adapters were removed using the skewer tool (v0.2.2, Jiang et al., 2014), and pairs of overlapping reads were joined using the PEAR tool (v0.9.11, Zhang et al., 2014). Quality control of the resulting sequences was carried out with the FastQC tool (v0.11.8). Before mapping the sequences of the reads from the MVs, an index of the *S. vesiculosa* M7^T^ genome was constructed with the bowtie2-build tool (v2.3.0, Langmead et al., 2019). Mapping of the MVs DNA fragments with *S. vesiculosa* M7^T^ genome was carried out using the bowtie2 tool (v2.3.0, Langmead et al., 2019). The above alignment resulted in a sam file for each sample mapped that stores the nucleotide sequence and characteristics of said sequence with a position in a reference genome. The sam files were converted into bam files, containing the same information in binary format using the samtools view tool (v1.11, Danecek et al., 2021). The FASTA file containing the *S. vesiculosa* M7^T^ genome was transformed into gtf format. The final file was transformed into a bed file by removing certain unnecessary information to reduce the size of the file. The previous bam files were then crossed with the bed file containing the *S. vesiculosa* M7^T^ genome annotation using the bedtools intersect tool (v2.29.2, Quinlan and Hall, 2010) to determine the genes found in the DNA fragments from MVs. For graphical representations, the bam format of the files of the DNA fragments of the MVs was changed. For that purpose, the bam files were sorted using the samtools sort tool (v1.11, Danecek et al., 2021) and subsequently indexed using the samtools index tool (v1.11, Danecek et al., 2021). Ordered, indexed, and aligned bam files of each sample were stacked using the samtools mpileup tool (v1.11, Danecek et al., 2021) and formatted to the graph coverage extension, to be read by the DNAplotter tool from Artemis (Sanger, United Kingdom; Carver et al., 2012).

### Statistics

Statistical analyses of the data were performed using StatGraphics XVII Version 17.2.07 (64-bit; StatGraphics Technologies, Inc., The Plains, Virginia). ANOVA tests were performed to analyze the mean differences between samples. The level of significance was set at *p*≤0.05 for all the tests.

## Results and Discussion

It has become evident that the type of bacteria (Gram-positive or Gram-negative) dictates the production of different types of MVs (Nagakubo et al., 2020). However, recent studies have shown that in Gram-negative bacteria, even members of the same species can secrete different types of MVs into the medium (Pérez-Cruz et al., 2013; Hagemann et al., 2014; Devos et al., 2017; Gill et al., 2019; Toyofuku et al., 2019). It is now clear that even careful isolation and purification lead to the recovery of a mixture of different types of MVs. Therefore, it is essential to identify the types of MVs a bacterium produces during its growth and the mechanisms governing MV formation that would influence the results of our subsequent studies.

### MV Secretion Depends on the Growth Phase of *S. vesiculosa* M7^T^

**Figure 1:**
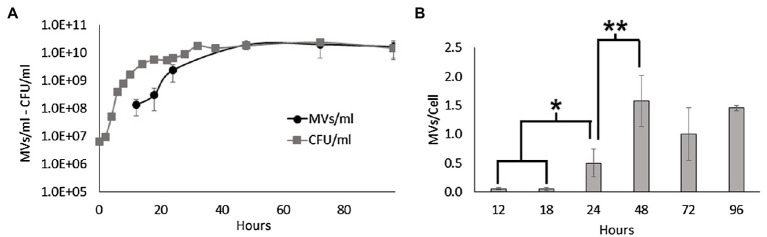
MV production during the growth of *Shewanella vesiculosa* M7^T^. **(A)** Growth curve of *S. vesiculosa* M7^T^ and the MV concentration during growth. Cell concentration was determined by Trypticase soy agar plate counting (CFU/ml). MV concentration was measured by flow cytometry with FM4-64 labeling, and events were extrapolated with the 100nm FluoSpheres^™^. **(B)** Graph representing the ratio or number of MVs secreted per cell along the growth curve. *n*=3, Statistical analysis was done using the ANOVA test, ^*^ and ^**^ *p*<0.05.

In this study, we investigated MV secretion during the growth of *S. vesiculosa* M7^T^ by high-resolution flow cytometry to directly enumerate FM4-64 stained MVs from culture supernatants (Wieser et al., 2014). For this purpose, *S. vesiculosa* M7^T^ was grown in 500ml of TSB at 15°C under agitation. At different time points, the number of viable cells and MVs were measured directly from filtered supernatants. Moreover, the number of MVs was normalized to the number of viable cells to measure the vesiculation rate (MV/cell) at each time point. As shown in Figure 1, the concentration of MVs increased during growth, but the vesiculation rate did not remain constant over time. During the first hours of incubation (12, 18h), the exponential growth of the cells and the MV concentration increased with a positive correlation (Figure 1A). However, during this time, the MV/cell ratio remained constant, with a value close to 0.05. It is important to note that *S. vesiculosa* M7^T^ growth halted between 18 and 24h of incubation, before accelerating again until reaching the stationary phase at approximately 32h. After 18h of incubation, there was a significant increase in the ratio of MVs/cell, while the vesiculation rate peaked at 48h with a stabilized value of 1.59 after that point. The transition from the exponential to the stationary phase, between 24 and 48h, was accompanied by the export of more MVs to the medium with significant differences in the vesiculation rate (Figure 1B), indicating that MV secretion depended on the growth phase.

Consistent with the literature, our results confirm that the concentration of MVs increases exponentially with cell density (Tashiro et al., 2010; Pérez-Cruz et al., 2021). Moreover, we found that the transition from the late exponential to the stationary phase prompted *S. vesiculosa* M7^T^ to export more MVs to the medium. MV formation depends on multiple factors such as growth phase, nutrient availability or environmental stressors. Transition from exponential to stationary phase is accompanied by changes in protein expression; in this process, misfolded or defective proteins and peptides from protein degradation may accumulate in the periplasmic space increasing the osmotic pressure and, thus, increasing vesiculation. In the same way, peptidoglycan fragments from cell wall turnover can also accumulate in the periplasmic space. MVs formation would release the pressure induced by the accumulation of these macromolecules (McBroom and Kuehn, 2007). In stationary phase, nutrients also begin to lack affecting MVs formation. Vesiculation is also strictly dependant of the outer membrane asymmetry, and this asymmetry is maintained by the VacJ/Yrb ABC system and a phospholipid transferase. The FUR regulator of the former is dependent on iron and in its absence hypervesiculation due to outer membrane asymmetry disruption (Roier et al., 2016). Similarly, sulfur depletion provokes NADPH overproduction, which is necessary for phospholipids production. Thus, the overproduction of phospholipids induces their accumulation in the outer membrane, disrupting its asymmetry and, finally, increasing MVs formation (Gerritzen et al., 2019). Different studies have reported that various stressors (oxidative stress, anoxia, UV radiation, antibiotics) promote prophage activation in prokaryotes which can act as inducers of membrane lysis and, eventually, MVs formation (Kageyama et al., 1979; Bamford et al., 1987; Beaber et al., 2004; Binnenkade et al., 2014; Fang et al., 2017).

### Nucleic Acid Content of MVs Varies During the Growth of the Bacteria

**Figure 2:**
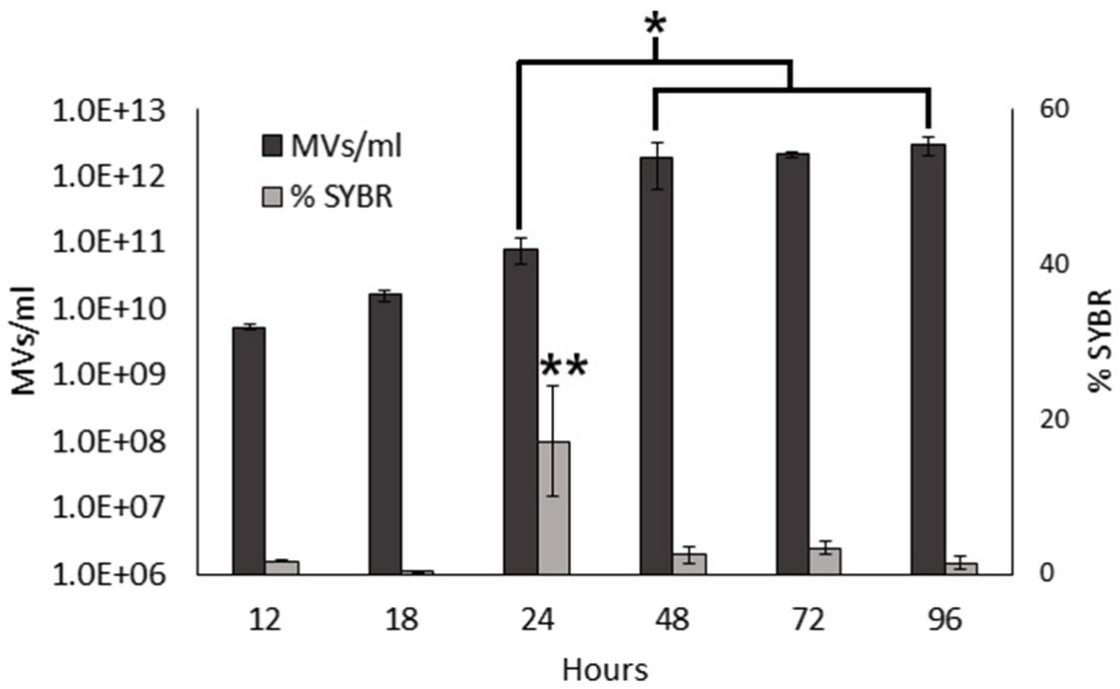
Nucleic acid content in *S. vesiculosa* M7^T^ MVs. Bar graph representing the total MV concentration (only labeled with FM4-64) and the percentage of nucleic acid-containing MVs (simultaneously labeled with FM4-64 and SYBR^™^ Gold) during growth. *n*=3, Statistical analysis was done using ANOVA test, ^*^ and ^**^ *p*<0.05.

Previous studies have shown that MVs from *S. vesiculosa* M7^T^ contain DNA mainly located inside O-IMVs (Pérez-Cruz et al., 2013). In this study, we aimed to determine if the nucleic acid content of MVs from *S. vesiculosa* M7^T^ varied during growth. We first isolated MVs at different stages of the growth curve, simultaneously labeled them with FM4-64 and SYBR^™^ Gold, and analyzed them by flow cytometry. FM4-64 with affinity for lipidic membranes was used to quantify MVs and SYBR^™^ Gold was used to detect nucleic acids inside them. The analysis of isolated MVs also showed that the concentration of MVs increased exponentially until 48h of growth and then stabilized up to 96h. Moreover, it is worth highlighting the marked increase in the concentration of MVs during the transition to the stationary phase between 24 and 48h (Figure 2), as observed before in the analysis in the supernatant. However, the percentage of SYBR^™^ Gold labeled MVs that contain nucleic acids did not increase similarly. Their percentage remained low for all the assayed times, with values between 0.34 and 3.38%. Moreover, they showed no significant differences between different time points, except at 24h when the percentage of SYBR^™^ Gold labeled MVs was very high (17%) and significantly different from all other points of the growth curve (Figure 2). These findings confirm that nucleic acid contents in MVs also depend on the growth phase, and suggest a possible induced cell lysis mechanism.

Assuming that nucleic acid-containing MVs are O-IMVs, their rise in concentration at 24h could be explained by the fact that O-IMVs are actively formed at this time point. This high percentage could imply a mechanism of O-IMV formation different from the blebbing mechanism previously described by us (Pérez-Cruz et al., 2013).

### Observation of Explosive Cell Lysis in *S. vesiculosa* M7^T^

Next, we aimed to investigate by TEM the appearance of cells and MVs during growth in order to detect events that would confirm an explosive cell lysis mechanism in *S. vesiculosa* M7^T^. This will allow us to explain the significant differences in the vesiculation rate during the transition to the stationary phase and the high percentage of SYBR^™^ Gold labeled MVs at 24h. For this purpose, TSB cultures of *S. vesiculosa* M7^T^ were centrifuged at different time points (12, 24, 48h) at high speed (44,000×*g*) to sediment cells and MVs simultaneously. Then, the samples were high-pressure fixed, freeze-substituted (FS), sectioned, and observed by TEM.

**Figure 3:**
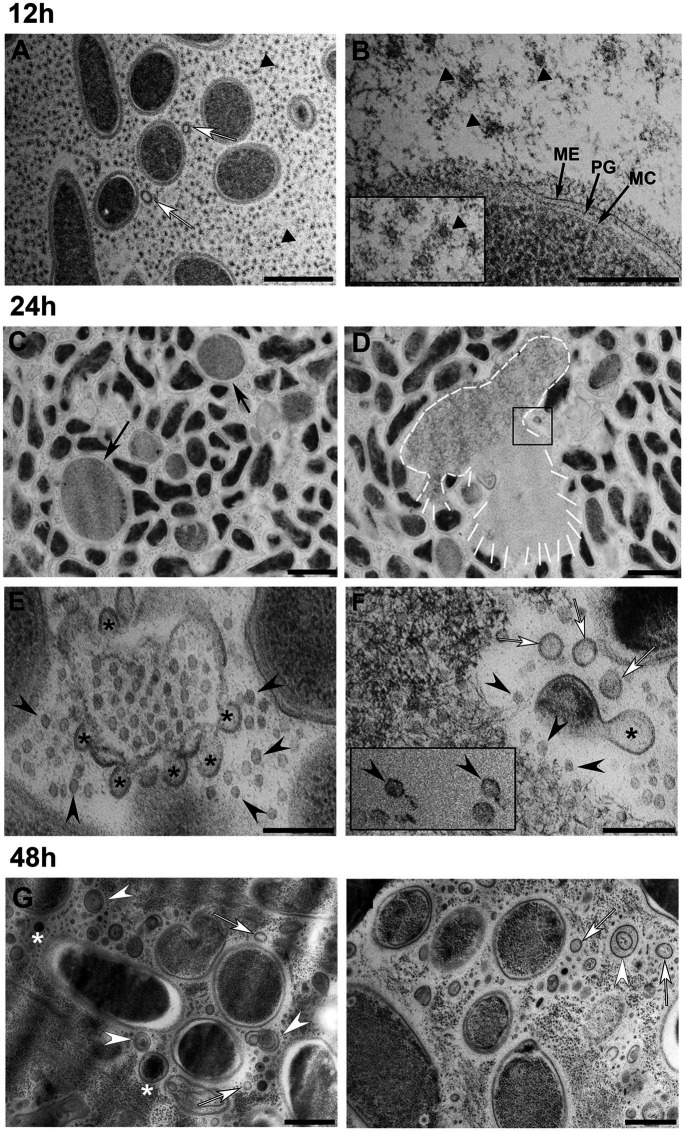
Transmission electron microscopy observation of *S. vesiculosa* M7^T^ cells and MVs at different times of growth. *S. vesiculosa* M7^T^ cells were collected from TSB liquid cultures by high-speed centrifugation and processed by high-pressure freezing and freeze substitution. The images are representative of the changes produced in cells and extracellular matter during growth of *S. vesiculosa* M7^T^. At 12h **(A,B)**, the cells appeared normal, and the extracellular space was occupied mainly by small round structures surrounded by fibrillar material (black triangles in A,B and inset in B). Some outer membrane vesicles (OMVs) were also observed (white arrows in A). At 24h **(C–F)**, being F an enlarged view of the inset in D, rounded and enlarged cells were visualized (black arrows in C). Cells exploding were observed (see white line-drawing in D), and reannealing membranes were detected at these points (asterisks in E–F) that led to the formation of OMVs (white arrows in F). At this time point, bacteriophages were observed (black arrowheads in E–F). At 48h **(G,H)**, the extracellular matter was complex containing the elements previously observed such as OMVs (white arrows in G) and complex vesicles with double-layered membranes (white arrowheads in G,H) or with electrodense material inside (asterisks in G). Scale Bars A-C,D, 1μm; B-E,F, 200nm; G,H, 500nm.

The appearance of the *S. vesiculosa* M7^T^ cells and extracellular matter markedly varied according to the time point at which they were observed (Figure 3). At 12h of incubation, most of the cells had regular appearances with well-defined envelopes (Figures 3A,B). The cells had a rod or round shape, depending on the section plane, with their cytoplasmic content being homogeneous, corroborating the characteristic stippling of the ribosomes. Moreover, the cell surfaces were covered by fine perpendicular fibers on the cell walls. The extracellular matter also consisted of vast amounts of round structures (Figures 3A,B, black triangles) that resembled small MVs (inset in Figure 3B). When viewed at higher magnification (Figure 3B), these structures seemed to derive from outer membrane fragments and dragged fibrillar material that surrounded the cells. These small MVs were regular in size with a mean diameter of 26nm. Larger MVs (around 70–100nm) having the characteristic appearance of OMVs were also observed, but much less frequently. The same fringe of fine fibers also surrounded the OMVs (Figure 3A, white arrows). These smalls MVs appeared to be derived from the outer leaflet of the cell’s outer membrane. Similar, uniformly distributed, small MVs (around 26nm in size) have already been observed by our research group in the extracellular matter of the cold-adapted Antarctic strain *Shewanella livingstonensis* NF22^T^, where they were related to cold-adaption (Frias et al., 2010); however, more in-depth analysis is required to determine if they could correspond to a different novel group of MVs with a specific function.

Next, we observed that at 24h, some of the *S. vesiculosa* M7^T^ cells lost their bacillary shape and acquired a rounded morphology. Moreover, their sizes became significantly larger than those of the other cells (Figure 3C, black arrows). A few cells also appeared exploded, liberating their cytosolic contents to the extracellular space (Figure 3D, white profile). TEM analysis corroborated all the morphological traits for explosive cell lysis described by Turnbull et al. (2016) as a biogenesis mechanism of bacterial MVs in *P. aeruginosa* biofilms. On the one hand, a small percentage of giant round cells were observed and exploding cells surrounded by membrane fragments with re-annealing tendencies were detected (Figure 3E). The higher resolution provided by TEM and HPF-FS clearly proved that the membrane fragments presented a bilayer structure with the same staining profile as that of the outer membranes of the cells. Interestingly, the regularly sized fragments at 24h gave rise to MVs with uniform diameters such as OMVs, named explosive-OMVs (EOMVs) by Toyofuku et al. (2019) to distinguish them from blebbing OMVs. In future studies, it would be enlightening to study the elements and mechanisms involved in this uniform membrane lysis and the tendency to re-circulize (Supplementary Figure S1).

The enlarged inset of the lysed cell (Figure 3F) showed 1,000s of tiny particles that possibly corresponded to the icosahedral heads of a bacteriophage (Figure 3F, black arrowheads). Occasionally, particles that possibly corresponded to complete bacteriophages were also observed. These particles had icosahedral heads and tails, which were challenging to visualize when dealing with thin sections (Figure 3F, black rectangle). We hypothesized that the explosion mechanism would be similar to that observed by Turnbull et al. (2016) in a *P. aeruginosa* strain using phase contrast, wide-field fluorescence, and f3D-SIM super-resolution microscopy. TEM observation clearly confirmed the explosion of a sub-population of cells in *S. vesiculosa* M7^T^ and the presence of phages at the point where explosion took place. Although bacteriophages have developed various lysis strategies for most Gram-negative phages, the key players in this process are the holins, endolysins, and spanins (Cahill and Young, 2019). In-depth analysis is required to confirm the presence of these proteins in *S. vesiculosa* M7^7^ and how they can affect the outer membrane breakage and, subsequently, the size of the formed MVs.

Explosive cell lysis is an essential mechanism for the generation of MVs of different types (Toyofuku et al., 2019). In our samples, we observed a mixture of shattered fragments of the outer membrane with curling and self-annealing tendencies at the points where the explosive cell lysis took place (Figures 3E,F, black asterisks). We observed that they also formed MVs (Figure 3F, white arrows; Supplementary Figure S1). Most of these re-annealed MVs were homogeneous in size with diameters of around 50nm with the typical structure of OMVs surrounded by a lipid bilayer and having the same profile as that of the outer membranes of the cells. Moreover, in 24h samples, the bacteriophages were highly concentrated at the lysis sites (Figures 3E,F) and scattered throughout the extracellular space. For our investigations, the samples of cells and MVs were prepared for TEM observation from agitated liquid cultures. Thus, bacteriophages, once liberated, became dispersed in supernatants. The lack of these cellular events in some of the 12h samples indicated the occurrence of explosive cell lysis between 12 and 24h.

Next, we observed how samples collected at 48h were significantly different from the other time points. Numerous MVs were observed scattered between the cells, without any trace of the original lysed cell. This observation could be attributed to the total disintegration of the lysed cell at this time point. Moreover, we observed that some of the MVs corresponded to OMVs (Figures 3G,H, white arrows). However, numerous MVs were seen containing electrodense material inside. In some cases, this intracellular material could be ribosomes having a granular appearance in the cell cytoplasm (Figures 3G,H, white asterisks). At 48h, many double-layered MVs were also seen, but most looked different from the O-IMVs previously described by our group. The membrane staining pattern observed in TEM did not allow us to distinguish if the two bilayers corresponded to the outer and inner membranes of the cells or if they arose from the same membrane (Figures 3G,H, white arrowheads). Moreover, many of the double-layered MVs observed at 48h did not show any electron dense material inside the inner layer, unlike the blebbing O-IMVs (Supplementary Figure S2). It is highly probable that recircularization of cell fragmented membranes formed the double-layered MVs after explosive lysis and not by extrusion of the outer membrane dragging along the inner membrane with a part of the cytoplasmic content. The name EOMVs was assigned to OMVs generated by explosive cell lysis (Toyofuku et al., 2019); we advocate that the double-layered O-IMVs formed after explosive cell lysis should be named explosive O-IMVs (EOIMV).

### Sequencing of Genome and MV DNA Confirmed a Bacteriophage Mediated Explosive Cell Lysis

**Figure 4:**
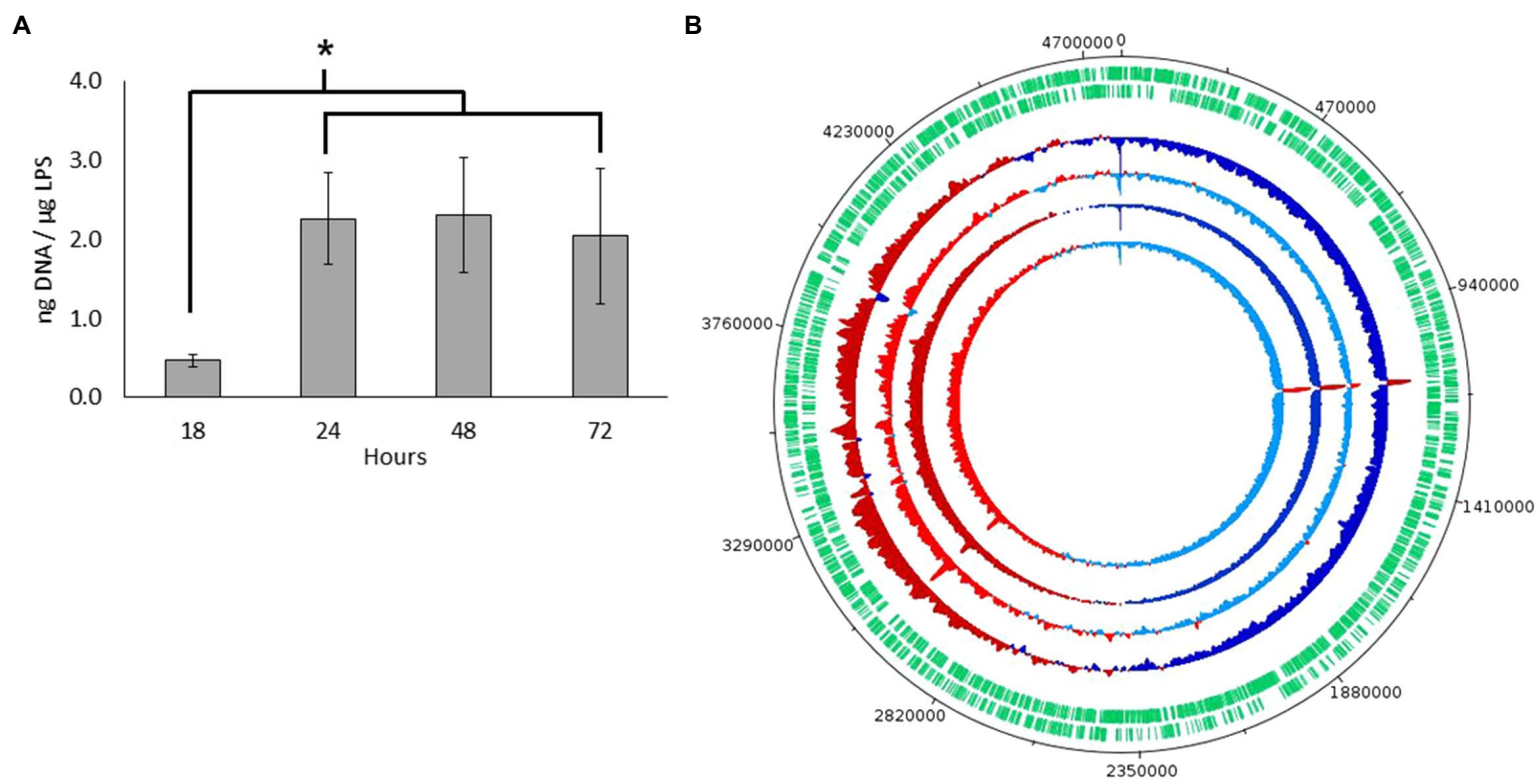
DNA characterization from *S. vesiculosa* M7^T^ MVs. **(A)** DNA concentration from S. vesiculosa M7^T^ MVs at different times of growth. *n*=3, ANOVA test, ^*^ *p*<0.05. **(B)** Circos plot for the MVs-DNA read count across each replicates show read counts at each base pair position. Positions that show greater than average read density are colored in red, and those with less than average density are colored in blue. Darker tones show fractions of fragments larger than 125bp, and lighter ones show fractions of fragments smaller than 125bp.

After confirming nucleic acids in MVs by high-resolution flow cytometry, we further quantified and characterized the DNA by sequencing. For quantification, DNA was extracted from three biological samples of MVs collected from *S. vesiculosa* M7^T^ treated with DNAse at different time points and quantified. DNA concentrations from the exponentially growing cultures (18h) were significantly lower than that of the samples collected at the transition from the late exponential to the stationary phase (24h). However, after this transition, the DNA concentrations remained constant during the stationary phase (Figure 4A). This observation corroborated findings from other studies that demonstrate that phage-induced MVs carry a higher amount of DNA and are more effective at horizontal gene transfer (Bearson and Brunelle, 2015; Andreoni et al., 2018; Crispim et al., 2019). Our results contradict the previous reports on *Streptococcus mutans* and *P. aeruginosa* (Liao et al., 2014; Bitto et al., 2017), which found more DNA association with MVs in the exponential phase than in the stationary phase. These last studies did not consider the explosive cell lysis mechanism in which DNA is likely to be entrapped in the recirculating membrane fragments, however more studies are needed to clarify these differences.

Next, to know which type of DNA was exported in MVs, we first sequenced the genome of *S. vesiculosa* M7^T^ to assemble and recircularize the *S. vesiculosa* M7^T^ chromosome with Illumina and Nanopore technology (NCBI accession number PRJNA723175). Then, we sequenced MV DNA to map the obtained fragments with *S. vesiculosa* M7^T^ chromosome., For this purpose the DNA from samples collected at the transition to the stationary phase (24h) were divided into two aliquots according to the size of the fragments. One of the aliquots contained DNA fragments bigger than 125bp, while the other contained fragments below 125bp. The two aliquots were sequenced separately. MVs-DNA sequencing showed that the MV DNA fragments represented the whole genome of *S. vesiculosa* M7^T^. Figure 4B shows the MV-DNA fragments’ representation along the *S. vesiculosa* M7^T^ genome, with the first half of the chromosome being represented below average (blue) and the second half being represented above average (red), similar to previous studies (Turnbull et al., 2016; Bitto et al., 2017) in different strains of *P. aeruginosa*. Other studies have reported the representation of only half of the genome in DNA isolated from MVs of the cyanobacteria *Prochlorococcus* (Biller et al., 2017). We also found a uniform representation of the fragments, although two over-represented peaks in 1.1 and 3Mb were detected. These fragments correspond to several coding sequences (CDS), even though most of the resulting proteins remain uncharacterized. Our analysis identified only a tyrosine-type integrase (RefSeq: WP_011637468.1) and a 23S ribosomal RNA that could be mapped to the obtained fragments.

The visualization of bacteriophage-like particles in our TEM analysis prompted us to initiate an *in silico* PHASTER tool-mediated (Arndt et al., 2016) search to identify prophage-like sequences in the *S. vesiculosa* M7^T^ chromosome. PHASTER showed one region of 37.1Kb with 52 protein-CDS, placed between 2.15 and 2.19Mb, that matched a phage-like structure. Moreover, the identified region showed high homology with the phiΦ18P phage previously described in the genus *Aeromonas* (Beilstein and Dreiseikelmann, 2008). The characteristics, localization, and structure of the identified prophage-like region are described in Supplementary Figure S3. TEM images and PHASTER tool confirmed that explosive cell lysis in *S. vesiculosa* M7^T^ was caused by a prophage activation. The morphology of viral particles visualized by TEM was comparable with phage phi18P, although virus isolation is needed to accurately define this new phage’s structural characteristics. Similar observations showing explosive cell lysis by prophage induction and secretion of different MVs were also reported in *Stenotrophomonas maltophila* (Devos et al., 2017).

### Apoptosis-Like Cell Death Was Observed in *S. vesiculosa* M7^T^

Prophage activation leads to DNA damage and activates a series of distinct cell death mechanisms referred to as ALD (Peeters and de Jonge, 2018). As growth arrest was detected in *S. vesiculosa* M7^T^ cultures at the transition from the late exponential to the stationary phase, we decided to identify the markers of ALD activation, such as the exposure of phosphatidylserine on the external face of the cell membrane or DNA fragmentation patterns.

**Figure 5:**
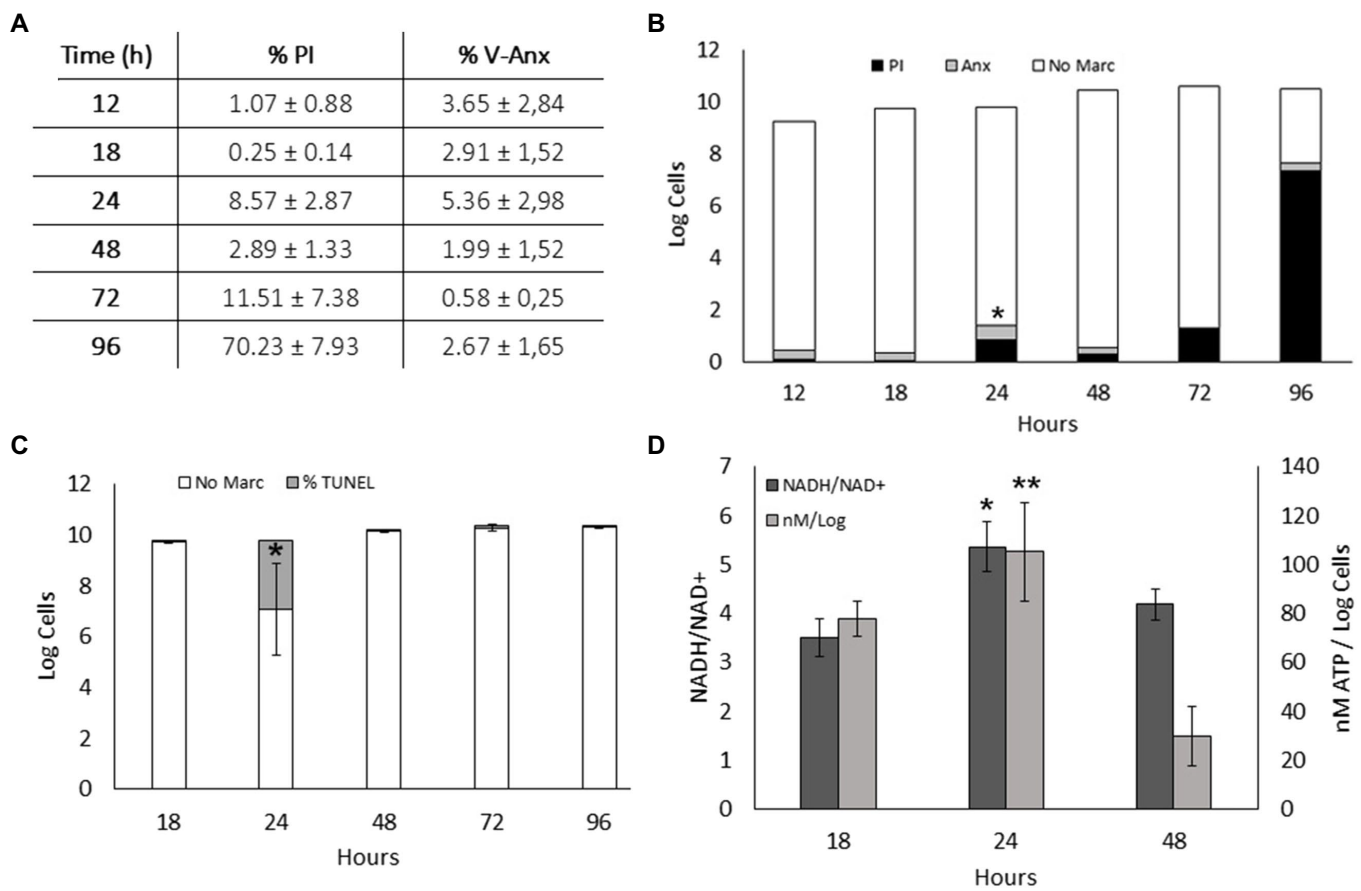
Apoptosis-like death in *S. vesiculosa* M7^T^ cultures. **(A)** Exposure of phosphatidylserine in *S. vesiculosa* M7^T^ cells at different time points of the growth curve was analyzed by flow cytometry after PI and Annexin V-FITC staining. **(B)** Bars graph representing percentage of PI (black) and Annexin V-FITC (gray) stained cells and unstained cells (white) at different time points of growth. *n*=3, ANOVA test, ^*^ *p*<0.05 of Annexin V-FITC stained cells. **(C)** Bars graph representing the percentage of TUNEL (gray) stained and unstained cells (white) at different times of growth. *n*=2, ANOVA test, ^*^ *p*<0.05. **(D)** Metabolic markers, ATP and NADH, in *S. vesiculosa* M7^T^ cells at different times of growth. *n*=3, ANOVA test, ^*^ and ^**^ *p*<0.05.

Exposure of phosphatidylserine was measured by flow cytometry of Annexin V-FITC- and PI-labeled *S. vesiculosa* M7^T^ cells collected at different time points of their growth. At 24h, significant differences in the percentage of Annexin V-FITC positive cells were detected compared with the other growth curve time points. Moreover, at the 24h time point, a non-significant increase in the percentage of PI marked cells indicated the death of a small portion of the population. This increase in PI staining corroborated the data of cell counts between 18 and 24h, reflecting the growth arrest, probably due to the phage lysis of the cells (Figures 5A,B). Next, the extent of DNA fragmentation was estimated by the TUNEL assay with the same samples. At 24h of incubation, 27% of the cells were positive for Alexa Fluor^™^ 488 compared with less than 1% at the other incubation times. This significant difference at 24h confirmed the phage replication-mediated DNA damage (Figure 5C). It is known that ALD processes require enzyme activation at the cost of highly energetic molecules like NADH or ATP, with a rise in the concentration of the reduced molecules compared with that of their oxidized counterparts. In our study, the NADH/NAD+ ratio and ATP concentration were significantly higher at 24h of incubation than for the samples collected from the exponential and stationary phases (Figure 5D).

The lack of apparent stressors (e.g., antibiotics, UV radiation, chemicals, or environmental stress factors) leads to explosive cell lysis, and MV secretion in *S. vesiculosa* M7^T^, unlike other strains (Toyofuku et al., 2012; Turnbull et al., 2016; Devos et al., 2017). Spontaneous induction of the lytic cycle can occur despite the stressful condition-mediated transition from the lysogenic to the lytic state of a prophage (Lwoff, 1953; Nanda et al., 2015). Several studies have demonstrated at a single-cell level that spontaneously occurring DNA damage under standard growth conditions induces an SOS pathway that in turn triggers the induction of prophages (Little, 1990; Nanda et al., 2014). Our investigation revealed distinct ALD mechanisms in *S. vesiculosa* M7^T^ cultures with significant differences in 24-h samples compared with other time points. We hypothesize that spontaneous phage lysis and activation of ALD processes lead to growth arrest at this time point. It is known that activation of a prophage can cause a bacterial community to induce the death of a fraction of their population (Little, 1990). Consequently, the SOS pathway activates the recA protein to induce a series of characteristic mechanisms of ALD (Maslowska et al., 2019).

### Separation of Different MVs by Flow Cytometry Sorting

Our previous studies demonstrated that *S. vesiculosa* M7^T^ produced OMVs and O-IMVs and that the latter contained DNA (Pérez-Cruz et al., 2013). The dragging of cytoplasmic components and cytoplasmic membranes and their outer membranes during O-IMV formation facilitated the incorporation of DNA in the O-IMVs. In this study, our TEM analysis confirmed the potential of *S. vesiculosa* M7^T^ to form various MVs during growth owing to bacteriophage-mediated explosive cell lysis. After cell explosion and lysis, self-annealing of membrane fragments could also explain the presence of double-layer MVs with nucleic acids inside. We aimed to separate the DNA-containing MVs by visually flow cytometry sorting and visualize them via Cryo-EM to determine their types. We isolated MVs from *S. vesiculosa* M7^T^ cultures at 24h owing to the significantly high percentage of double-labeled MVs. For flow cytometry-based sorting, we established one channel to collect MVs labeled with FM4-64 and another channel to collect those labeled with FM4-64 and SYBR^™^ Gold. This experiment was carried out twice, and in each one, between 1–1.5 million events were separated. After sorting, each separated sample was ultracentrifuged at 100,000*g*, resuspended in a minimal amount of sterile water, and plunge-frozen (PF) for observation by Cryo-EM.

**Figure 6:**
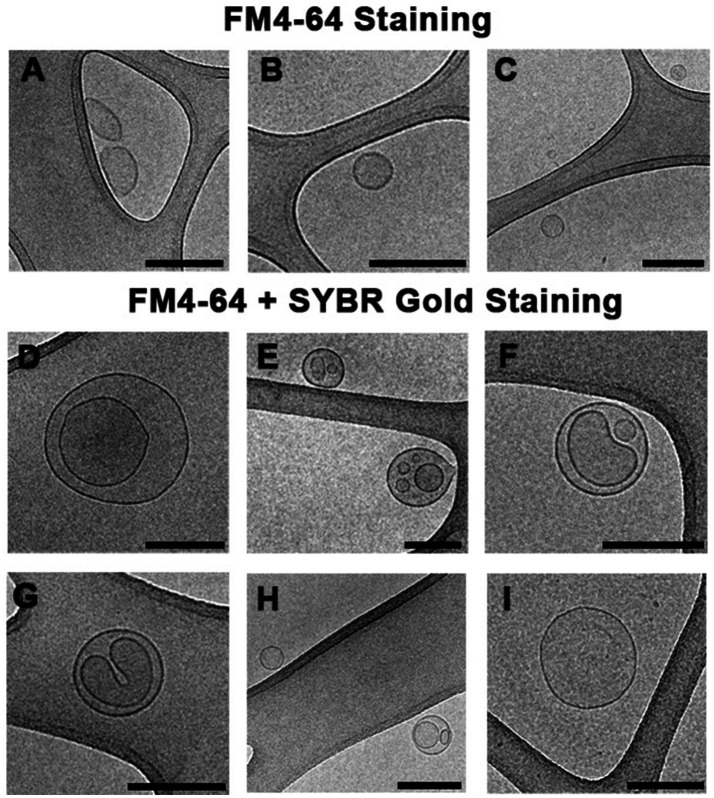
Cryo-electron microscopy images of MVs from *S. vesiculosa* M7^T^ separated by flow cytometry sorting. MVs were isolated from TSB liquid cultures of *S. vesiculosa* M7^T^ at 24h of incubation and were submitted to sorting by flow cytometry. Cytometry events were separated into two channels. Events marked only with FM4-64 were collected in one channel, and events marked with FM4-64 and SYBR^™^ Gold in another one. Subsequently, they were ultracentrifuged and cryofixed by Plunge Freezing. The images are representative of the different types of MVs observed by Cryo-EM. Images **(A–C)** show that events labeled with only FM4-64 corresponded mostly (90%) to single-layered MVs probably corresponding to OMVs. Images **(D–I)** show events marked with both fluorochromes and corresponded predominantly to double-layered MVs (70%); some of them have the same structure described for outer-inner membrane vesicles **(D)**, while others showed strange shapes with an external layer and more than one vesicle inside **(E–H)**. A lower percentage of MVs (30%) marked with both fluorochromes were visualized as one-layered MVs **(I)**. Bars, 200nm.

We successfully separated the MVs despite the challenging nature of the experiment (Figure 6). We demonstrated that the events labeled with only FM4-64 corresponded mostly to single-layered OMVs (90% of visualized MVs; Figures 6A–C). However, events marked with both fluorochromes corresponded predominantly to double-layered MVs (70% of visualized MVs), with several distinguishable types (Figures 6D–H). Based on the Cryo-EM structure, we hypothesize that some double-layered MVs in *S. vesiculosa* M7^T^ could correspond to the previously described O-IMVs (Figure 6D). However, many of them have a different appearance, with more than one vesicle inside the external layer and strange shapes of the inside content (Figures 6E–H). A percentage of MVs marked with both fluorochromes (30%) were visualized as one-layered MVs, confirming that during the process of membrane re-annealing after cell lysis, OMVs also entrap nucleic acids (Figures 6H,I).

## Conclusion

In summary, our novel findings highlight the growth phase-dependent production of different amounts and types of MVs by *S. vesiculosa* M7^T^. Moreover, we demonstrate how a part of the population incurred spontaneous induction of phage-mediated explosive cell lysis in the transition from the late exponential to the stationary phases, and led to the secretion of different types of vesicles. After phage lysis, a different type of O-IMV (EOIMV) was detected with structural differences compared with the blebbing O-IMVs previously identified in *S. vesiculosa* M7^T^. High-resolution flow cytometry is a valuable tool to monitor the production of different MVs during growth; here, it facilitated the first separation of MVs based on their nucleic acid content. Even so, there remains a clear need to implement improved bacterial MV separation techniques to advance our knowledge of MVs. This study shows that prophage activation can be a determinant factor in MV formation and should be taken into consideration when studying Gram-negative bacteria MVs.

## Data Availability Statement

The data presented in the study are deposited in the NCBI repository, accession number PRJNA723175. Direct link is (www.ncbi.nlm.nih.gov/bioproject/PRJNA723175). The names of the repository/repositories and accession number(s) can be found in the article/Supplementary Material.

## Author Contributions

NB and EM contributed to the conception and design of the study, conducted most experiments, performed the statistical analysis, and wrote the manuscript. JC assisted in the design and performance of the flow cytometry experiments. LD assisted in the performance of TEM and Cryo-EM experiments. All authors contributed to the manuscript, and all have read and approved the submitted version.

## Funding

This work received funding from grant CTQ2014-59632-R to EM and scholarship BES-2015-074582 to NB, both from the Ministerio de Economia y Competitividad, Spain. Grant 2014SGR1017 was awarded by the Departament d’Innovació, Universitats i Empresa from the Autonomous Government of Catalonia. The funders had no role in the study design, data collection and analysis, decision to publish, or preparation of the manuscript.

## Conflict of Interest

The authors declare that the research was conducted in the absence of any commercial or financial relationships that could be construed as a potential conflict of interest.

## Publisher’s Note

All claims expressed in this article are solely those of the authors and do not necessarily represent those of their affiliated organizations, or those of the publisher, the editors and the reviewers. Any product that may be evaluated in this article, or claim that may be made by its manufacturer, is not guaranteed or endorsed by the publisher.

## Supplementary Material

### Importance of the Study

It is known that bacteria secrete various types of vesicles with different morphological and functional characteristics. Studies to characterize the different types of vesicles are important to harness their multiple potentials in the field of biology and biotechnology. In this study, we used a model vesiculating bacteria to characterize the different kinds of vesicles secreted. We show that the amount and type of vesicles depends on the growth phase and on the activation of a phage-mediated explosive cell lysis leading to the release of a heterogeneous pool of single- and double-layered vesicles. After phage lysis, a different type of double-layered vesicle was detected with structural differences compared with the blebbing model previously identified in this strain. For the first time, an attempt has been made to separate bacterial vesicles based on their nucleic acid content. However, improvement and implementation of vesicle separation techniques should be a priority to advance knowledge of vesicles and their biological functions.
