# Supplementary material for: Phage-Mediated Explosive Cell Lysis Induces the Formation of a Different Type of O-IMV in Shewanella vesiculosa M7T
Source: Front Microbiol. 2021 Oct 8;12:713669. doi: 10.3389/fmicb.2021.713669 (PMC8529241; doi:10.3389/fmicb.2021.713669)
Supplement: Supplementary file 1 [file Presentation_1.pdf]

Supplementary Materials.

Growth phase and explosive cell lysis induction determine the type of membrane vesicles secreted by *Shewanella vesiculosa* M7^T

Nicolás Baeza[1], Lidia Delgado[2], Jaume Comas[3], Elena Mercade[1]

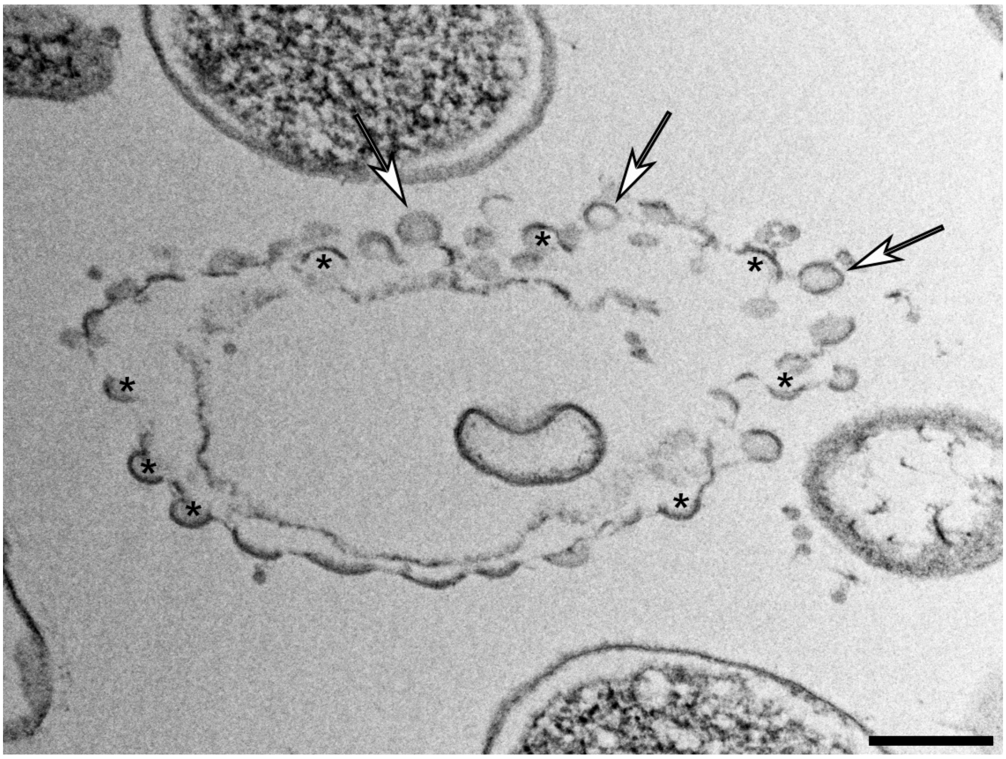

Fig S1. TEM observation of *S. vesiculosa* M7^{T} cells collected at 24 h from TSB liquid cultures by high-speed centrifugation and processed by HPF-FS. Image show how OM fragments tend to curl, re-anneal and finally form OMVs. White arrows point out OMVs. Asterisks are located at points where re-annealing fragments from the OM are observed. Bar 200 nm.

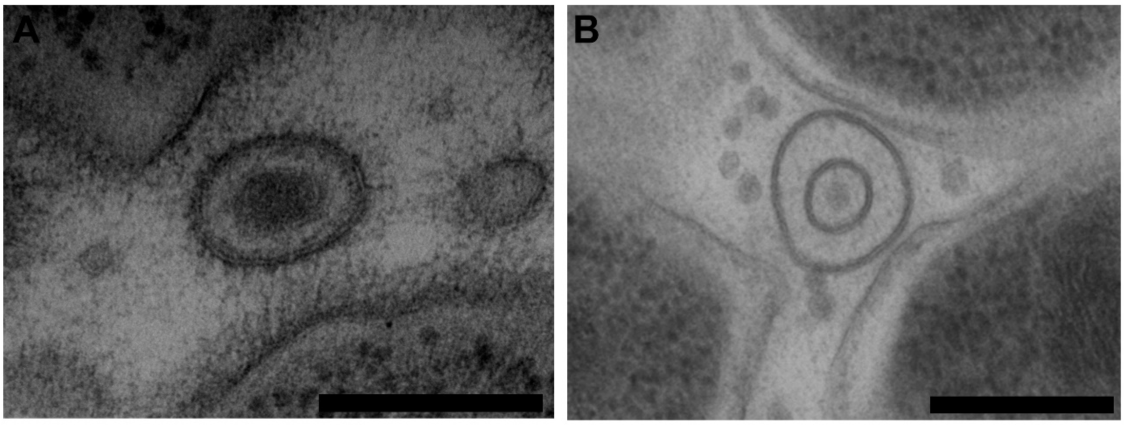

Fig S2. TEM observation of *S. vesiculosa* M7^{T} cells and supernatant collected by high-speed centrifugation and processed by HPF-FS. (A) Image shows an O-IMV with a two-layer structure and an electrodense material inside the inner membrane. **(B)** Image shows an O-IMV with a two-layer structure, but no content was observed inside the inner membrane. Bars 200 nm.

A

Region	Length (Kb)	Completeness	Score	# Total Proteins	Region Position	Most Common Phage	%GC
1	37.1	Intact	150	52	2150154 - 2187271	PHAGE_Aeromo_phiO18P_NC_009542(23)	42.41

B

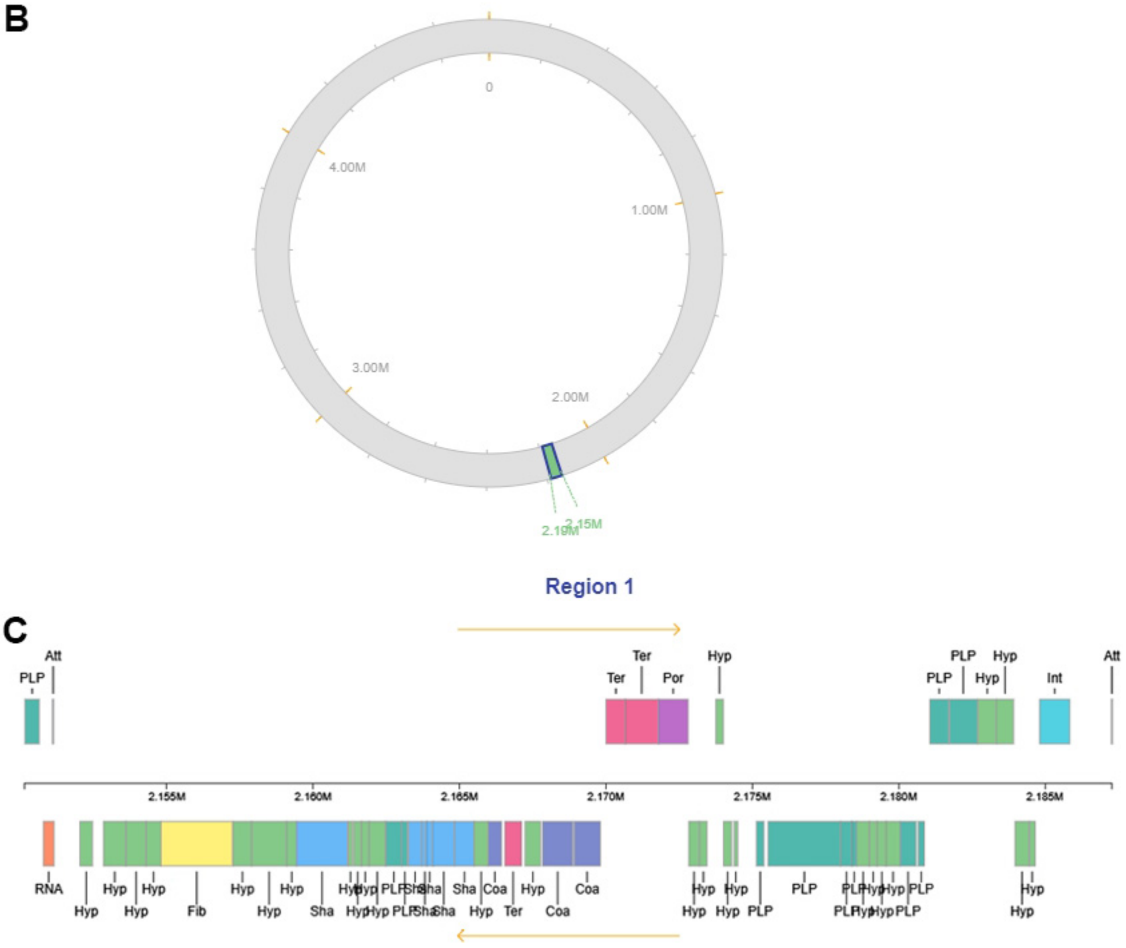

Fig S3. ***In silico*** **detection of prophages in** ***S. vesiculosa*** **M7^{T} genome by PHASTER tool. (A)** Characteristics of the identified prophage. **(B)** Graphic localization of the identified prophage in the *S. vesiculosa* M7^{T} chromosome. **(C)** Graphic representation of the structure, position and functions of the identified genes in the prophage region.
